# Effectiveness of the P3-speller in brain–computer interfaces for amyotrophic lateral sclerosis patients: a systematic review and meta-analysis

**DOI:** 10.3389/fneng.2014.00012

**Published:** 2014-05-01

**Authors:** Mauro Marchetti, Konstantinos Priftis

**Affiliations:** ^1^Department of General Psychology, University of PadovaPadova, Italy; ^2^Laboratory of Neuropsychology, IRCCS San Camillo HospitalVenice, Italy

**Keywords:** brain–computer interface, amyotrophic lateral sclerosis, P3-speller, systematic review, meta-analysis

## Abstract

A quarter of century ago, Farwell and Donchin (1988) described their mental prosthesis for “talking off the top of your head.” This innovative communication system, later named P3-speller, has been the most investigated and tested brain–computer interface (BCI) system, to date. A main goal of the research on P3-spellers was the development of an effective assistive device for patients with severe motor diseases. Among these patients are those affected by amyotrophic lateral sclerosis (ALS). ALS patients have become a target population in P3-speller (and more generally in BCI) research. The P3-speller relies on the visual sensory modality, and it can be controlled by requiring users to actively move their eyes. Unfortunately, eye-movement control is usually not spared in the last stages of ALS, and, then, it is definitively lost in the case of complete paralysis. We reviewed the literature on ALS patients tested by means of P3-speller systems. Our aim was to investigate the evidence available to date of the P3-spellers effectiveness in ALS patients. To address this goal, a meta-analytic approach was adopted. The pooled classification accuracy performance, among retrieved studies, was about 74%. This estimation, however, was affected by significant heterogeneity and inconsistency among studies. This fact makes this percentage estimation (i.e., 74%) unreliable. Nowadays, the conclusion is that the initial hopes posed on P3-speller for ALS patients have not been met yet. In addition, no trials in which the P3-speller has been compared to current assistive technologies for communication (e.g., eye-trackers) are available. In conclusion, further studies are required to obtain a reliable index of P3-speller effectiveness in ALS. Furthermore, comparisons of P3-speller systems with the available assistive technologies are needed to assess the P3-speller usefulness with non-completely paralyzed ALS-patients.

## INTRODUCTION

**FIGURE 1:**
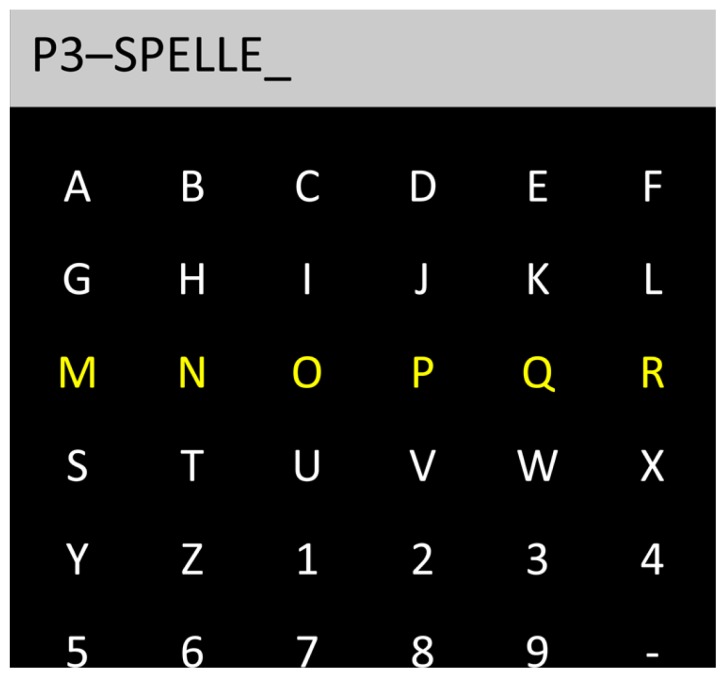
**Schematic representation of a P3-speller BCI.** Letters and numbers are displayed in a *N* × *N* matrix (6 × 6 resulting in 36 symbols in the present example). Columns and rows are randomly flashed for a short time (i.e., the third row from the top in this matrix). Users have to focus their spatial attention on the target stimulus (e.g., letters) that they want to select. When the selection is correct, a larger P300 is elicited when the columns and the row containing the to-be-selected target stimulus are flashed. Usually, the classified stimuli are displayed on the top of the matrix.

Twenty-six years ago, Farwell and Donchin (1988) first described a spelling system that exploited event-related potentials (ERPs) for selecting alphanumeric stimuli on a screen, later called the P3-speller. Participants were shown a 6 × 6 matrix of symbols (i.e., letters and functions; **Figure 1**). The rows and the columns of the matrix were randomly flashed, and the participants were required to focus their visuospatial attention on a specific target symbol.

Farwell and Donchin (1988) reported that a rather distinct ERP (i.e., P3 component; for a review about P3, see Polich, 2007) was elicited by the flash occurring in the combination of columns and rows, in which the attended letter was positioned. Moreover, they investigated the possibility to detect the P3 associated with the target letter, by processing offline the ERPs (event-related potentials) through algorithms for signal detection. The first ERP-based brain–computer interface (BCI) was born. Following this seminal study, the P3-speller paradigm has become the most studied one, in the BCI domain (Cecotti, 2011). The success of the P3-speller is mainly due to three reasons. First, it relies on electroencephalography (EEG), which is a non-invasive and cheap technique that can be moved according to the patients’ needs (e.g., it can be used at their bedside or it can be implemented on a wheelchair; Daly and Wolpaw, 2008). Second, the P3-speller is an ERP-based BCI, and, thus, it does not require a long training period with respect to BCIs guided by sensorimotor rhythms (SMRs) and slow-cortical potential (SCPs; Birbaumer, 2006). Third, the P3-speller paradigm permits users to select among several symbols/commands. For instance, Townsend et al. (2010) have proposed a 9 × 8 symbols’ speller, whereas the SCP- and SMR-based BCIs provide users with fewer choices (the most common is a binary choice; Birbaumer, 2006).

The main goal in BCIs’ research is to offer a new channel of communication and control, with particular regard to patients affected by severe motor diseases (Wolpaw et al., 2002). Because of the nature of the illness, ALS patients have been the main target population in BCI studies (for reviews, see Kübler and Birbaumer, 2008; Moghimi et al., 2013). Voluntary muscle control is progressively affected in ALS; thus, in the later stages of the disease the patients become totally paralyzed. The first cause of death in ALS is respiratory failure (Radunovic et al., 2013). Survival can be prolonged in those of the patients who decide to have respiratory support (i.e., tracheotomy or long-term mechanical ventilation) and the feeding tube (Dreyer et al., 2013). ALS evolves toward the locked-in syndrome (LIS), a condition in which patients remain conscious but they lose their ability to voluntary control most of their muscles (Smith and Delargy, 2005). For instance, ALS-LIS patients may become unable to express their opinions and decisions on important questions regarding their clinical treatment, or their living and biological wills. Hence, effective BCIs could have an enormous impact on the life of ALS-LIS patients, by permitting them to communicate and interact with their environment. Prior to entering the LIS condition, however, ALS patients are still able to communicate, by exploiting their residual motor abilities. Eye-movements are usually one of the last voluntary movements in ALS patients before they reach the complete LIS condition (i.e., CLIS), in which no voluntary muscle control is retained (Murguialday et al., 2011). The reliable control of a muscle (e.g., eye-muscle control) is generally used as a channel for interaction between the patients and their environments. Communication, however, is usually limited to binary yes/no answers to respond to caregivers’ and clinicians’ questions, whereas the P3-speller offers a higher number of possible choices to ALS patients (i.e., *N* × *N* choices). Then, when reliable control is reached, users do not depend on other people’s questions for communicating, but they can spontaneously “speak out” words or sentences. For these reasons, several researchers have proposed the P3-speller as a potential solution for communication problems of ALS patients (Münβinger et al., 2010; Townsend et al., 2010; Manyakov et al., 2011; Li et al., 2011). Unfortunately, the P3-speller is useless for ALS-CLIS patients, because their visual modality is completely impaired (i.e., paralysis of the eyes, dryness of the cornea; Murguialday et al., 2011). Indeed, other sensory modalities must be exploited, with ALS-CLIS patients, in order to develop effective BCIs (e.g., the acoustic modality; Sellers and Donchin, 2006; Kübler et al., 2009). A further limit of the P3-speller is that it requires users to focus their gaze on the to-be-selected target stimulus (e.g., letters, numbers, etc.). In fact, two independent studies on healthy participants have shown that the P3-speller performance relies on the possibility of the users to move their eyes for focusing on the target stimulus (Brunner et al., 2010; Treder and Blankertz, 2010). This represents a further problem with ALS patients, because several oculomotor dysfunctions accompany the progression of the illness (e.g., ophthalmoplegia, defective pursuit eye-movements, saccadic movements’ impairment, nystagmus; for a comprehensive review, see Sharma et al., 2011). As a consequence, the P3-speller may be useful only for those ALS-LIS patients who retain sufficient eye-muscle control, and who cannot control communicative prostheses that require limb movements. After focusing on the patients who are in this latter condition, however, there is one main point to be considered. When eye-muscle control in an ALS-LIS patient is sufficient for controlling a P3-speller, it can be reasonably hypothesized that the same patient could control an eye-tracker for augmentative and alternative communication (AAC; Beukelman et al., 2011). Eye-trackers do not need time for electrodes montage and require short calibration time, even with infants (Gredebäck et al., 2009). Furthermore, eye-trackers have accurate, fast and reliable classification performances (Holmqvist et al., 2011), and can be satisfactorily used for hours by ALS patients (e.g., for 300 min; Spataro et al., 2013). Unfortunately, no direct comparison between P3-spellers and eye-tracking spelling systems is available in the literature, to date.

In a recent editorial in which the new horizons of BCI were discussed, Sellers (2013) explicitly formulated the following question: “*Can people with somewhat compromised visual ability benefit from a visual BCI?*” With the present meta-analysis, we aimed to partially address this question (i.e., P3-speller is only one of the available visual BCIs), by focusing on the effectiveness of P3-speller BCIs in ALS patients.

## MATERIALS AND METHODS

### SEARCH STRATEGIES AND SELECTION CRITERIA

In June 2013 we performed a search on the Pubmed database. We searched the terms “P3-/P300-speller,” or “brain-computer interface(s),” or “BCI,” or “brain–machines interface(s),” or “BMI,” or “man–machines interface(s),” or “direct brain interface(s),” or “mental prosthesis/-es” in combination with each of the following terms: “amyotrophic lateral sclerosis,” or “ALS,” or “motor neuron disease,” or “MND.” We searched the reference list of retrieved papers to identify additional relevant articles. Only studies in English were considered for the present systematic review. Original studies reporting P3-speller tests with ALS patients were selected. The choice of a performance’s measure that permits a clear and direct comparison across BCIs is a question of theoretical debate in the literature (Dal Seno et al., 2010; Yuan et al., 2013). Thus, for the meta-analysis we avoided the use of information transfer rate (ITR), which is often misreported in literature (Yuan et al., 2013). We identified, instead, the classification accuracy (CA) as our target measure. CA is defined by the percentage of correct target selection with the P3-speller. CA is a common index of performance reported among BCI studies, and offers a clear idea about BCI systems’ effectiveness in target classification. On the contrary, CA does not give any information about the system speed for selecting commands. ALS patients, however, have declared the need of a BCI with CA above 90%, as their priority, followed by the communication speed issue (Huggins et al., 2011). In fact, a fast but unreliable BCI would be useless for paralyzed patients who cannot communicate through other AAC systems.

### ENDPOINTS AND STATISTICAL ANALYSIS

For the meta-analysis, we extracted from each study: the CA and its relative measure of variability around the mean (e.g., standard deviation, standard error of the mean, etc.), the year of publication, the chance level (CL = 100/*N* of matrix symbols) of CA associated to each P3-speller, and the sample size. Each time an ALS patient was tested with more than one P3-speller paradigm, only the best CA was chosen. The CA, defined as the percentage of correct target selection, was used as endpoint for addressing the question of P3-spellers’ effectiveness in ALS. The reported measures of variability around the averaged CA of each study (i.e., standard error and standard deviation) were used to compute the 95% confidence intervals around the effect size measure (i.e., the row CA). Because there was a wide variability of experimental designs and of goals among the retrieved studies, there might have been different effect sizes underlying the studies in our meta-analysis. Thus, we calculated the pooled CA by using a random-effects model, assuming that the effect sizes of the studies that actually were performed represented a random sample of the potential real ones (Borenstein et al., 2009; Cumming, 2012). Heterogeneity and inconsistency in the results of the selected studies were assessed by means of Cochran’s *Q* and *I*^2^ tests, respectively (Higgins et al., 2003). To address the possible publication bias (e.g., the fact that studies with non-significant results are less likely to be published), we computed the Kendall’s tau rank correlation with continuity correction and the Egger’s regression tests (Rothstein et al., 2005). Analyses were performed using Comprehensive Meta-Analysis (v. 2.2.064) and the “metafor” package (Viechtbauer, 2010) of the R statistic software (v. 3.0.1).

## RESULTS

**FIGURE 2:**
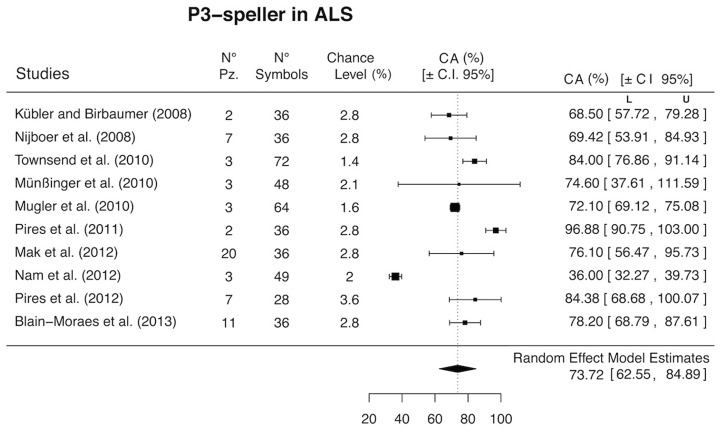
**Forest plot of P3-speller accuracy in ALS.** The percentage of classification accuracy (CA %) and associated confidence intervals (CIs) are reported for each study. L, lower limit of the confidence interval; U, upper limit of the confidence interval; N° pz, number of patients included in each study; N° symbols, number of symbols displayed in the matrices presented in each study; chance level, the percentage of symbols, in each study, that could have been correctly selected by chance.

The systematic search resulted in 454 non-duplicated records. Of these, 440 articles were excluded because P3-spellers were not tested with ALS patients. Four studies described only single cases of ALS patients (Escolano et al., 2010; Sellers et al., 2010; Aloise et al., 2011; Manyakov et al., 2011), and, thus, they could not be included in the meta-analysis. The averaged CA of the four single cases tested with P3-spellers was 71.82%. Finally, ten eligible studies were identified and were entered in the random-effects model analysis (**Figure 2**).

**FIGURE 3:**
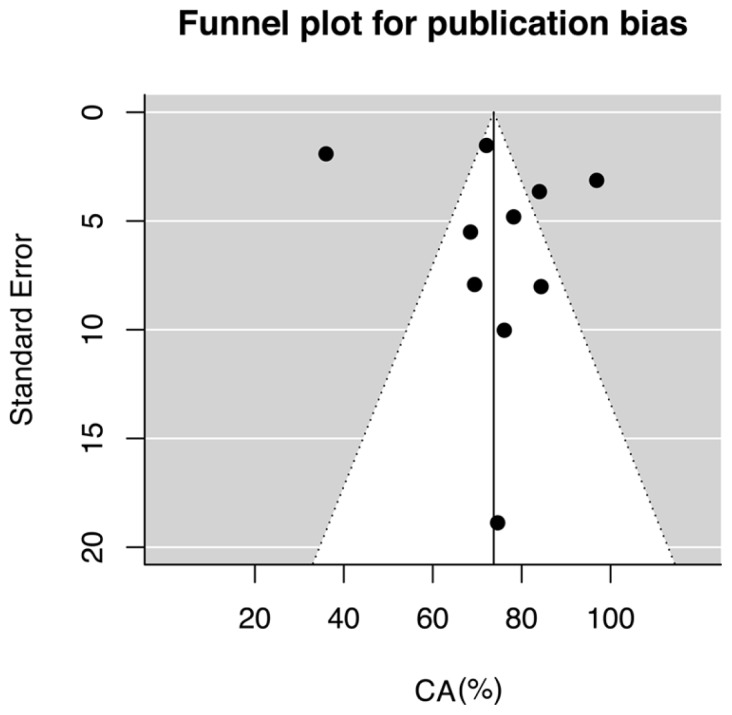
**Funnel plot of P3-speller accuracy in ALS.** Distribution of studies to the left and to the right of the median line of the funnel plot is rather symmetric, suggesting that there is no evidence of publication bias.

The estimated CA of these pooled studies was 73.72% (95% CI, 62.55 to 84.89). The estimation of this result, however, is limited by the significant heterogeneity and inconsistency among the considered studies (*Q* = 399.41, *p* <0.001; *I*^2^ = 95.71). Then, a meta-regression on all eligible studies was performed, in which the publication’s year was used as moderator. The CA did not significantly increase as a function of time (i.e., publication year; *B* = 6.67, C.I. 95%: -18.8 to 32.13; *Q*_model_ = 0.26, *p* = 0.61). The funnel plot for publication bias resulted symmetrical to visual inspection (**Figure 3**). The statistical analyses performed for testing the publication bias were both non-significant [Egger’s test, *t*(8) = 0.822, *p*(2-tailed) = 0.43; Kendall’s τ = -0.35, *p*(2-tailed) = 0.15].

## DISCUSSION

In the present meta-analysis we investigated the available evidence of the effective use of P3-speller BCIs with ALS patients. The pooled CA of P3-speller with ALS patients was about 74%. This estimated result was affected, however, by huge inconsistency among the analyzed studies. This significant heterogeneity is probably due to differences in study designs (e.g., differences in: sample sizes, number of sessions, classification methods, etc.). Hence, the observed wide variability limits the possibility of safely considering the overall estimation.

Even by assuming that the estimation of the present meta-analysis is correct, some further considerations are necessary. It is clear that a 74% level of CA is far above the chance level (usually lower than 3% with P3-spellers). But, at the same time, 74% is considerably lower than the 90% CA desired by ALS patients (Huggins et al., 2011). Whether a 74% level of CA could be sufficient for everyday use of the P3-speller, remains an empirical question. Of course a 74% level of CA might be considered as satisfactory for patients with no other means of communication. Note, however, that if ALS patients have no other means of communication, they would be probably unable to perform the eye-movements required for controlling the P3-speller.

In most of the studies included in the present meta-analysis, there was a limited number of experimental sessions (e.g., one or few days of testing), which took place often in non-ecological settings (i.e., not at patients’ home, which is the last goal for an assistive technology; Kleih et al., 2011). There is only one peer-reviewed report, in which an ALS-LIS patient reached satisfactory long-term control of a P3-speller (for more than 2 years, and at home), with an accuracy above 80% (Sellers et al., 2010). It could be pointed out that the overall CA computed in the present meta-analysis is biased, and the computation could have underestimated the real performance of ALS patients using the P3-speller. Nonetheless, both the tests that we performed on the publication bias were not significant. It is true that the publication bias tests may be underpowered, as a consequence of the small number of studies retrieved for the analysis. But if a publication bias is present, it is more probable that studies that failed to find successful performance were not published, than vice versa. Cases of unsuccessful P3-speller use would have resulted only in a lower CA estimation.

The usefulness of P3-spellers with ALS patients has to be discussed under the light of a further consideration. The performance estimated in the meta-analysis was obtained from samples of ALS patients with sufficiently spared oculomotor functions; otherwise meaningful control of a P3-speller is not possible (Brunner et al., 2010; Treder and Blankertz, 2010). When eye-movement control is spared, a word processor could be controlled even by means of eye-trackers. Pannasch et al. (2008) have described four ALS-LIS patients performing an eye-tracker copy-spelling task, with the possibility of correcting misspelled letters. ALS-LIS patients accomplished the task with 100% accuracy in each tested session, with an average speed of 17 selections per minute. The eye-tracker technique does not require montage of sensors on the user, and it requires only few minutes of calibration for being ready-to-use. Moreover, Spataro et al. (2013) have described a group of 30 ALS patients who satisfactorily used an eye-tracker device for about six hours per day, mainly for communicating with their caregivers. Before thinking to move the P3-speller from the labs to ALS patients’ houses, one should consider whether P3-spellers offer any advantage to ALS patients with respect, for example, to the advantages of eye-trackers. To our knowledge, however, there are no peer-reviewed studies in which the ALS patients’ performance, in using a P3-speller versus an eye-tracker, has been directly compared.

The findings of the present meta-analysis do not bring clear evidence of P3-speller usefulness with ALS patients. New studies, with larger samples of ALS participants -for increasing power-, with better specified inclusion/exclusion criteria, with detailed assessment of residual eye-movement control, and with clearly and comprehensively reported descriptive statistics are required in order to reach a reliable estimation of P3-speller effectiveness. We would like to underline that our findings are limited to the P3-speller interface, and cannot be in any way generalized to other BCI systems. The eye-movement problem related to the P3-speller is nowadays well known (Brunner et al., 2010; Treder and Blankertz, 2010). Some alternative visual BCIs guided by evoked potentials, and relying on covert spatial attention orienting (i.e., no eye-movement required), have been tested with ALS patients (Lim et al., 2013; Marchetti et al., 2013). Nonetheless, when the visual modality is no more exploitable for ALS-patients, the chances of communication by means of a BCI are entrusted on other sensory modalities (e.g., acoustic or tactile) or on EEG signals other than ERPs (for a review on eye-gaze independent EEG-based BCIs, see Riccio et al., 2012). Despite the huge interest that the P3-speller has received, and on the basis of the evidence from the present meta-analysis, the early hypothesized goal of translating P3-spellers into a mental prosthesis for everyday use (Farwell and Donchin, 1988), for ALS patients, has not been met yet.

## Conflict of Interest Statement

The authors declare that the research was conducted in the absence of any commercial or financial relationships that could be construed as a potential conflict of interest.
